# *Dejian *mind-body intervention improves the functioning of a patient with chronic epilepsy: a case report

**DOI:** 10.1186/1757-1626-2-9080

**Published:** 2009-11-24

**Authors:** Agnes S Chan, Sophia L Sze, Mei-chun Cheung, Joseph MK Lam, Dejian Shi

**Affiliations:** 1 Neuropsychology Laboratory, Department of Psychology, The Chinese University of Hong Kong, Shatin, Hong Kong SAR, China; 2 Integrative Neuropsychological Rehabilitation Center, The Chinese University of Hong Kong, Shatin, Hong Kong SAR, China; 3 Institute of Textiles and Clothing, The Hong Kong Polytechnic University, Hunghom, Hong Kong SAR, China; 4 Department of Surgery, The Chinese University of Hong Kong, Shatin, Hong Kong SAR, China; 5 Henan Songshan Research Institute for Chanwuyi, Henan, China

## Abstract

**Background:**

This is a case report that illustrates the effect of the *Dejian *Mind-Body Intervention (DMBI) on a 22-year-old chronic epileptic male (onset at age two) suffering from severe cognitive impairments as a result of a serious seizure two years ago. The DMBI is a healing program developed for modern lifestyle based on the traditional Chinese *Shaolin Chanwuyi *healing approach.

**Case Presentation:**

Through a four-month treatment in which he adopted the DMBI specified vegetarian diet, applied an herbal remedy, and practiced Natural *Dan Tian *Breathing (a type of mind-body exercise), the patient showed significant improvements in language, memory, attention, behavioral initiation, emotional control, social functioning, and overall quality of life. In addition, the DMBI has a positive effect on his brain electrophysiological activities, as indicated by his suppressed delta power (slow wave) and enhanced alpha power (fast wave), and his elevated cordance value (an index associated with cerebral perfusion) in the left frontal and temporal regions. Such neural activity alteration was in line with his observed cognitive improvements.

**Conclusion:**

These results provide evidence to support the therapeutic effect of the DMBI and its potential clinical application on treating chronic neurological patients.

## Background

The *Dejian *Mind-Body Intervention (DMBI) was developed upon the traditional Chinese *Shaolin *Buddhist medical practice known as *Chanwuyi *by the last and the first author. *Chanwuyi *is a unique healing approach built upon the principle of unblocking the *Qi *and clearing the bodily orifices. The DMBI is a holistic intervention that aims to enhance the health of both mind and body. Cumulative empirical evidence revealed the connection between the mind and the body, and the therapeutic effects of many mind-body interventions improved mental and physical health problems [1]. Clinical observations on the DMBI showed that patients with different diseases including late-stage cancer, stroke or brain tumor demonstrated various degrees of improvement, and a randomized controlled trial also showed positive results [2].

**Figure 1:**
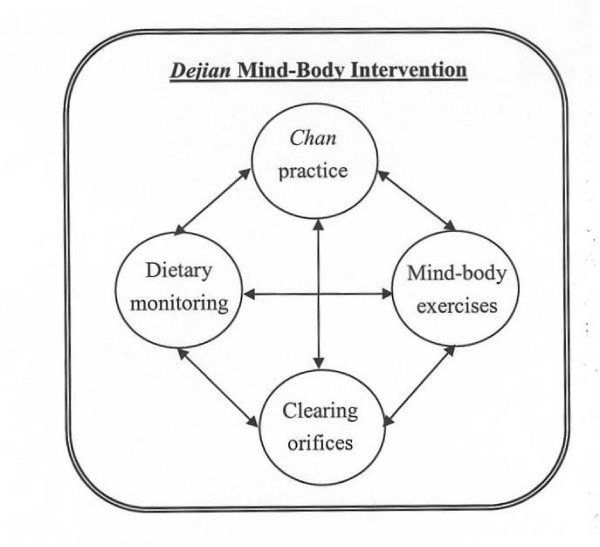
**Schematic representation of the DMBI treatment model**.

The DMBI consists of four interconnected components: (1) *Chan *practice, (2) mind-body exercises, (3) dietary monitoring, and (4) clearing the orifices; which aims to improve physical and psychological health (Figure 1). Some empirical studies have been done to examine the effect of DMBI [2-4], and found that the DMBI was more effective compared with a brief cognitive-behavioral therapy in improving the mood of individuals with moderate to severe depressive mood after one-month intervention [2]. The DMBI also helped an adolescent with Asperger's syndrome improve self-control, psychosocial functioning and overall quality of life [3]. Another study showed the effect of the specially formulated herbal remedy (a method to clear the major orifice - the nose) on enhancing the activity of the frontal-lobe and anterior cingulate cortex of the brain of normal individuals [4]. In addition, some recent quantitative EEG results for examining the neural-basis for the treatment effectiveness of the mind-body exercises of the DMBI, namely, the *Dan Tian *breathing, showed that this type of breathing increased the left-right alpha asymmetry and theta coherence of the cortex [4]. Since left-right alpha asymmetry is associated with positive emotion [4] and theta coherence is associated with increased attention [5], these results suggested that this type of breathing may increase positive mood and attentiveness. We applied the DMBI on a young adult patient suffering from epilepsy since two years of age and who sustained severe brain damage and cognitive impairments after a serious seizure two years ago. Some encouraging results are described in the present report.

## Case presentation

### Medical History

The patient, CK, is a 22-year-old Chinese male suffering from epilepsy since the age of two and is currently working in a sheltered workshop. He had no relevant family medical history and did not smoke or drink alcohol. He had persistent and frequent seizure episodes at about four times a month. He underwent magnetic resonance imaging (MRI) scan on his brain in 2005, and was found to have left mesial temporal sclerosis and left frontal proencephalic cyst. Results of video electroencephalogram (EEG) also revealed a focal onset in the left frontal-temporal region. Neuropsychological assessments indicated that CK was moderately impaired in verbal memory (with immediate recall of 6 out of 16 words and rapid forgetting after 10 minutes) as measured by the Hong Kong List Learning Test (HKLLT) [6], and moderately impaired in naming common objects as measured by the Boston Naming Test (BNT; with a score of 18/30) [7]. He also showed signs of frontal-lobe dysfunction, e.g., difficulty in retrieving learnt verbal information and mildly impaired verbal organization and inhibitory control. Nevertheless, he was generally intact in general intelligence, visual attention and memory functions. In May 2005, CK received left hippocampectomy, after which his seizure frequency was substantially reduced to six episodes a year.

In August 2006, CK suffered from a severe seizure resulting in further deterioration in his language, attention, memory, initiation of behavior, emotional control and motor functions. In the following eight months, he underwent treatments including hyperbaric oxygen therapy, acupuncture, and physiotherapy. Nevertheless, his cognitive functions except motor ability remained severely impaired. He had to live with his mother's assistance and could only work in a sheltered workshop. CK began to receive DMBI two years after the incident and one year after all other interventions ended. At baseline before receiving the DMBI, we assessed CK on his cognitive functions using the BNT, Chinese Mini-Mental Status Examination (CMMSE) and Multilingual Aphasia Examination (MAE) Token Test; but he was not able to perform any of the tasks given his severely impaired cognitive functions. He could only produce one word at a time, and was mostly non-responsive to testing instructions but occasionally elicited a delayed response after repeated prompting.

### Dejian Mind-Body Intervention

CK was recommended a specific vegetarian diet with abstinence from meat, seafood, ginger, garlic, green onion and spicy foods. CK used an herbal remedy provided by the Institute of Chan Herbal Medicine under the Chanwuyi Foundation Limited (registered charity organization in Hong Kong) and manufactured under strict Good Manufacturing Practice standards.  He applied the remedy intranasally three times daily (10 ml per time) to clear the nasal orifice. He also practiced Natural *Dan Tian *Breathing (NDTB) two to three times daily. During NDTB, an individual puts his/her hand(s) gently on the *Dan Tian *(i.e., about half inch below the navel) while breathing in and out naturally. The individual also passively and relaxingly observes the *Dan Tian *region during inhalation and observes the nose during exhalation.

### Baseline Measures

#### Overall function

Before the DMBI, CK's overall functioning was measured using the Functional Independence Measure (FIM) [8] and the Functional Status Rating System (FSRS) [9] with his mother as informant. According to the FSRS, CK demonstrated severe impairment in communication and cognitive abilities (with a score of 9/28 and 5/20 respectively), and moderate impairment in social functioning (with a score of 11/16). His score on the FIM also indicated that he was completely dependent requiring maximal assistance from his mother on communication and social-cognitive aspects (Table 1). CK seldom demonstrated spontaneous or imitated speech. He had difficulty in understanding simple instructions, which has to be repeated at least five times to elicit his delayed (at least one minute) response. He had severe memory impairment and forgot what was told after half a minute, and had difficulty remembering familiar routes and direction. He had flat affect and rarely verbally expressed emotion for others. In face of difficulties, he became irritable and had temper tantrums (Table 2).

**Table 1:** The Changes in Overall Functioning of Patient CK from Baseline to Post-Treatment

Scale	Baseline	Post-treatment	Improvement(%)
The Functional Independence Measure			
Self-care	37/42	39/42	5
Sphincter control	14/14	14/14	N/A
Mobility	21/21	21/21	N/A
Locomotion	14/14	14/14	N/A
Communication	5/14	7/14	40
Social and cognitive abilities	4/21	7/21	75
Total score	95/126	102/126	7
The Functional Status Rating System			
Self-care	34/36	35/36	3
Mobility	18/20	19/20	6
Communication	9/28	13/28	44
Psychosocial Adjustment	11/16	13/16	18
Cognitive function	5/20	10/20	100
Total score	77/120	90/120	17

**Table 2:** Detailed Changes in the Cognitive Functions of Patient CK from Baseline to Post-Treatment

	Baseline	Post-treatment
Expressive language	Less than 3 meaningful word per day	More than 10 meaningful words per day
	No spontaneous speech most of the time	Some spontaneous speech
	Very little imitated speech	Frequently imitated the mother's speech
Comprehension	Difficulty in executing single instruction	Executed 3- to 4-step complex instructions
	No response to request or execution after repeated instructions	Occasionally executed instructions without repetition
	Reacted after at least 10 sec	Sometimes reacted within 3 sec
	Could not distinguish left from right	Distinguished left from right
Memory	Instantly forgot other people's words	Remembered at least 3- to 4-step complex instructions
	Could not recognize familiar routes and direction	Can recognize familiar routes and direction
Attention and awareness	Usually paid no attention to things and persons in the environment	Increased awareness of the environment
	Maintained attention to copy 3 to 4 words	Maintained attention to copy a maximum of 20 words
Emotional expression and control	Had temper tantrums once or twice a week on average	Less than one temper tantrum a week on average
	Flat affect	Increased smiling
	Did not share joy with others	Shared joy with the mother
Eye contact	Dull, unfocused, little eye contact	Alert, focused, had eye contact during communication
Interpersonal relationship	Rarely cared about other people	Actively cared about the health/safety of other people
	Resented body contact from the father, distant relationship with the father	Allowed touching by the father, improved relationship with the father
	Did not seek help when in need	Sought help when in need

#### Quality of life

CK's mother rated her son 3/10 in the Quality of Life Index (QL Index) [10] total score, indicating a low quality of life. Due to his cognitive impairments, he rarely helped out with household chores and had to be closely monitored when travelling to and from the sheltered workshop. His social life was restricted to recreational activities with inmates or occasional family gatherings. CK had poor physical health and had influenza and fever about once a month. He had temper tantrums and his relationship with his father was distant. All these in conjunction created great stress for his mother.

#### Quantitative electroencephalogram

**Figure 2:**
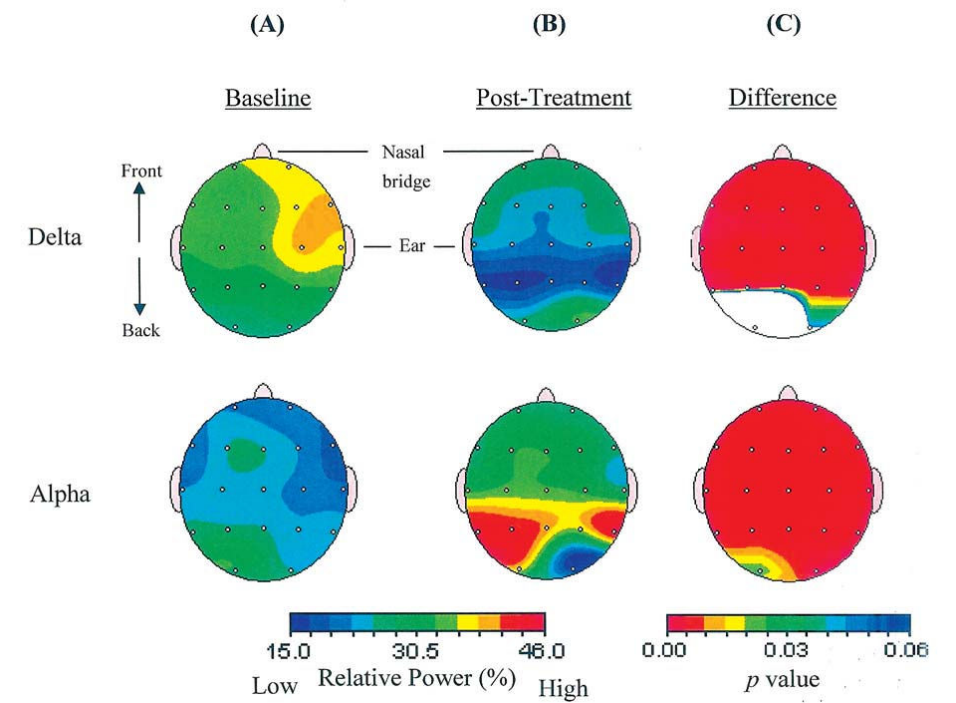
**Changes in EEG relative power before and after intervention**. Level of relative power at delta and alpha frequency band at (**A**) baseline and (**B**) post-treatment. (**C**) Results of paired t tests comparing relative power between baseline and post-treatment time, where red color indicates p < .005.

Quantitative EEG data were recorded from CK and analyses were focused on relative delta (1-4 Hz) and alpha (8-12 Hz) power. Excessive delta power is often related to brain disorders, e.g., epilepsy [11], traumatic brain injury [12]. Higher alpha power is often related to attentional and memory processing [13], and inhibitory control [14]. As shown in Figure 2A, CK demonstrated a relatively higher percentage of delta power and lower percentage of alpha power, which spread from the anterior (frontal) to the post-central (temporal and parietal) brain regions.

**Figure 3:**
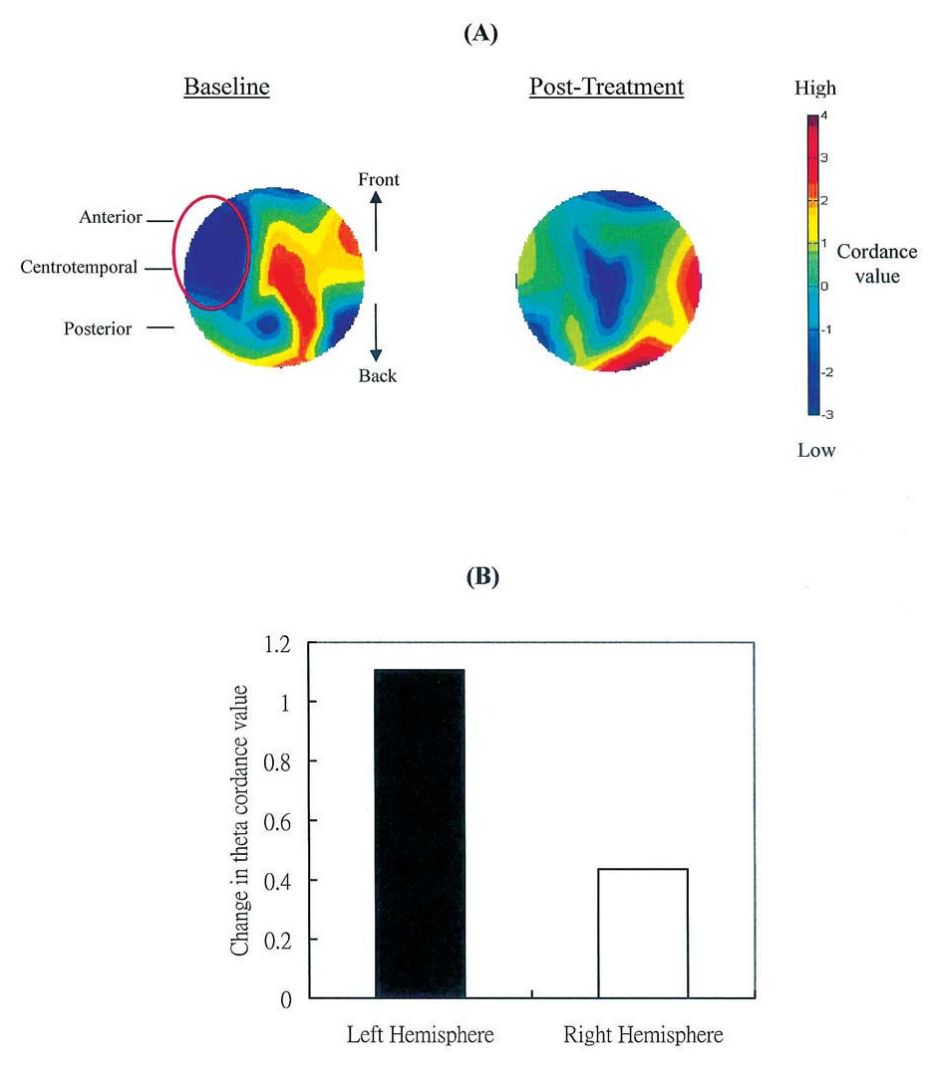
**Changes in theta cordance before and after intervention**. (**A**)Topographic maps showing cordance at baseline and post-treatment. (**B**) Changes in mean theta cordance averaged across the anterior, centrotemporal and posterior regions in the left and right hemisphere. Positive values indicate increased cordance value after treatment.

We further analyzed the EEG data using the cordance index, which was developed by Leuchter and colleagues [15] and proposed as an indirect measure of brain perfusion. Brain perfusion reflects the amount of blood flow to the brain tissues. Higher perfusion suggests higher metabolism in a region. The cordance value in theta (4-7 Hz) was found to be moderately correlated with cerebral perfusion as measured by Positron Emission Tomography [15]. Results showed that CK's theta cordance in the left anterior and centrotemporal regions were relatively lower than other scalp regions (Figure 3A, circle in red), suggesting lower perfusion in the two regions. His reduced theta cordance at baseline was consistent with his MRI findings in 2005 showing brain damage in his left mesial temporal and frontal lobe.

### Treatment Outcomes

#### Overall function

After the four-month DMBI, CK demonstrated a functional improvement of 40-100% in communication and cognitive abilities in the FIM and FSRS (Table 1). CK demonstrated three times more spontaneous and imitated speech, and could execute up to 4-step complex instructions (Table 2). He could react instantaneously at times after the first presentation of instructions. In addition, he demonstrated enhanced awareness of the surroundings, sustained attention, improved emotional expression and control (e.g., eliciting appropriate affect and being less irritable in face of difficulty), better social interaction with increased eye contact, and expressed concern and acceptance of his family members.

#### Quality of life

CK's quality of life improved from 3/10 to 7/10 (a 133% improvement) on the QL Index. His most noticeable improvement was in physical health; he had not fallen sick since receiving the DMBI. His temper was better controlled and he began to maintain a more harmonious relationship with his father. With his improved cognitive functioning, he was able to help out with household chores and independently travel to and from the sheltered workshop. Given CK's improvement, his mother reported less burden and stress in taking care of CK.

#### Quantitative electroencephalogram

Figures 2A and 2B show the topographic maps of relative delta and alpha power at pre- and post-treatment respectively. Results of paired *t-*tests using Neuroguide 2.1.9, showed a significant reduction in delta and increment in alpha (p < .005) covering frontal, temporal and parietal brain regions after treatment (Figure 2C). In addition, CK's initially suppressed cordance values at the left anterior and centrotemporal regions at baseline elevated substantially by 1.45 and 0.9 units respectively after the four-month intervention (Figure 3A). A similar increment (0.97 unit) was also observed in the left posterior region. The overall cordance increment in the left hemisphere was significantly different from 0 [t(2) = 6.40, p = .024], whereas the increment in the right hemisphere was non-significant [t(2) = .62, p = .60] (Figure 3B).

## Discussion

This is a case report on the effect of a four-month *Dejian *Mind-Body Intervention on a chronic epileptic patient, administered two years after the onset of his severe cognitive impairments. By adopting a special vegetarian diet, daily application of an herbal nasal drop, and practising Natural *Dan Tian *Breathing, the patient showed considerable improvements in his cognitive abilities, which in turn significantly improved his quality of life. qEEG results also indicated that the patient's brain activities were altered in a positive direction with reduced delta power and enhanced alpha power, and increased cordance value in the left hemisphere which implicated enhanced cerebral perfusion. These changes in brain activities may underlie the observed improvements in his cognitive functions.

While the concept of "window of recovery" in the rehabilitation process of neurological patients has been well understood that functional recovery mostly occurs within the first year after the accident, the patient in this present study began to receive the DMBI two years after the severe epileptic attack, and one year after cessation of other treatments when his recovery process already plateaued for a year. After practicing the DMBI for a short four-month period, the patient was observed to have improved in a number of important cognitive functions including language, memory, attention, behavioral initiation and emotional regulation, as well as positive neurophysiological changes. The patient's functional improvements not only enhanced his quality of life, but also alleviated the burden and stress of his primary care-taker. Given that it is clinically unusual to observe spontaneous recovery 2 years after the accident, the improvements noted in this patient is conceivably related to the DMBI. The easy-to-practice and economical characteristics of the DMBI and its quick therapeutic effect as shown in the present study implicates its possible clinical application on neurological patients.

The significant delta suppression and alpha elevation may underlie the behavioral improvements observed in the patient. Previous studies revealed that excessive delta power and reduced alpha power is often associated with brain disorders/dysfunction [11,12], and higher alpha power was correlated with better higher cortical functions (including attention, memory and inhibitory control) [13,14]. The patient's suppressed delta and elevated alpha suggested positive changes in his neuro-electrophysiological condition, which was in line with his improved attention, memory, and emotional control. In addition, his increased theta cordance in the left anterior and centrotemporal regions may reflect enhanced cerebral perfusion in his left frontal and temporal regions where MRI and video EEG showed abnormal brain structure and activity. His treatment-induced cordance elevation suggested enhancement of brain functions that coincided with his behavioral changes. The frontal-lobe is an important brain structure that mediates speech output, sustained attention, initiation of behaviors, appropriate expression and control of emotions and behaviors; and the temporal lobe plays a major role in mediating learning and memorization of new information. The patient's functional improvement covered those cognitive aspects shown in Table 2. Some recent empirical evidence also supported the effect of the DMBI on enhancing frontal-lobe activities in normal adults (A. Chan, PhD, et al., unpublished data, May 2009) and adults with depressed mood [2]. Together, these suggest that the DMBI has a potential effect on enhancing brain activity and its associated functions in normal individuals as well as patients with brain disorders.

In addition to functional improvements, the DMBI, as a holistic intervention approach on the health of the mind and the body, also improved the physical health of the patient. While the patient used to get sick at least once a month, he has not been sick since receiving the DMBI. These findings were consistent with previous clinical observations demonstrating improved health in patients receiving the DMBI and empirical evidence on improvements in bowel function in a group of community-dwelling adults [2]. These accumulating evidence suggest that the DMBI is an effective treatment to improve the physical health of patients non-specific to any particular disease.

## Conclusion

In sum, this case report provides evidence to support the therapeutic effect of the DMBI on improving cognitive functions, neurophysiological state and physical health, which in turn enhanced the quality of life of the patient and his care-giver. Since the treatment was administered on the patient two years after the onset of cognitive impairments and after the patient had undergone other treatments, the observed improvements is unlikely to be due to spontaneous recovery. Given the low cost of the DMBI compared with other treatments and the DMBI showed effect in a short period of time, more long-term study of the therapeutic effect of the DMBI is warranted. Future studies focusing on whether the effect can be sustained over time, and whether a more long-term treatment program may lead to even more enhanced effect, will be helpful.

## Competing interests

The authors declare that they have no competing interests.

## Authors' contributions

Author ASC provided the intervention for the patient, who was referred by JMKL. DS formulated the intervention. ASC and SLS helped with the neuropsychological and EEG assessment, statistical analyses and data interpretation. ASC also supervised drafting of the manuscript. All authors contributed to and approved the final manuscript.

## Consent

Written informed consent was obtained from the patient for publication of this case report and accompanying images. A copy of the written consent is available for review by the Editor-in-Chief of this journal.

## References

[B1] Jacobs GD Clinical applications of the relaxation response and mind-body interventions J Altern Complem Med 2001 7 S93 S101 10.1089/107555301753393850 11822640

[B2] Chan AS Cheung MC Tsui WJ Sze SL Shi D *Dejian *mind-body intervention on depressive mood of community-dwelling adults: a randomized controlled trial Evid Based Complement Alternat Med Advance Access published May 27, 2009, doi:10.1093/ecam/nep043 19474241 10.1093/ecam/nep043 PMC3136532

[B3] Chan AS Sze SL Shi D Traditional Chinese mind-body exercises improve self control ability of an adolescent with Asperger's disorder J Psychol Chin Soc 2008 9 225 239

[B4] Chan AS Cheung MC Sze SL Leung WW Shi D An herbal nasal drop enhanced frontal and anterior cingulate cortex activity Evid Based Complement Alternat Med 2009 in press 10.1093/ecam/nep198 PMC3140066 19996154

[B5] Sauseng P Klimesch W Schabus M Doppelmayr M Fronto-parietal EEG coherence in theta and upper alpha reflect central executive functions of working memory Int J Psychophysiol 2005 57 97 103 10.1016/j.ijpsycho.2005.03.018 15967528

[B6] Chan AS Hong Kong List Learning Test 2006 2 Hong Kong: Department of Psychology and Centre for Neurocognitive Function Enhancement, The Chinese University of Hong Kong

[B7] Cheung RW Cheung MC Chan AS Confrontation naming in Chinese patients with left, right or bilateral brain damage J Int Neuropsychol Soc 2004 10 46 53 10.1017/S1355617704101069 14751006

[B8] Granger CV Hamilton BB McDowell I, Newell C The functional independence measure Measuring Health: A Guide to Rating Scales and Questionnaires 1996 New York: Oxford University Press 115 120

[B9] Forer SK McDowell I, Newell C The functional status rating system Measuring Health: A Guide to Rating Scales and Questionnaires 1996 New York: Oxford University Press 76 78

[B10] Spitzer WO McDowell I, Newell C The quality of life index Measuring Health: A Guide to Rating Scales and Questionnaires 1996 New York: Oxford University Press 405 409

[B11] Altay EE Fessler AJ Gallagher M Correlation of severity of FDG-PET hypometabolism and interictal regional delta slowing in temporal lobe epilepsy Epilepsia 2005 46 573 576 10.1111/j.0013-9580.2005.08204.x 15816953

[B12] Thatcher RW North DM Curtin RT An EEG severity index of traumatic brain injury J Neuropsychiatry Clin Neurosci 2001 13 77 87 11207333 10.1176/jnp.13.1.77

[B13] Klimesch W EEG alpha and theta oscillations reflect cognitive and memory performance: A review and analysis Brain Res Rev 1999 29 169 195 10.1016/S0165-0173(98)00056-3 10209231

[B14] Klimesch W Sauseng P Hanslmayr S EEG alpha oscillations: The inhibition-timing hypothesis Brain Res Rev 2007 53 63 88 10.1016/j.brainresrev.2006.06.003 16887192

[B15] Leuchter AF Uijtdehaage SHJ Cook IA O'Hara R Mandelkern M Relationship between brain electrical activity and cortical perfusion in normal subjects Psychiatr Res: Neuroimaging 1999 90 125 140 10.1016/S0925-4927(99)00006-2 10482384

